# Tonabersat Inhibits Retinal Inflammation After Hypoxia–Ischemia in the Neonatal Rat

**DOI:** 10.3390/ijms26167996

**Published:** 2025-08-19

**Authors:** Jack Jonathan Maran, Alice McDouall, Justin M. Dean, Joanne Davidson, Odunayo O. Mugisho

**Affiliations:** 1 Buchanan Ocular Therapeutics Unit, Department of Ophthalmology, New Zealand National Eye Centre, University of Auckland, Auckland 1023, New Zealand; jmar555@aucklanduni.ac.nz; 2 Department of Physiology, University of Auckland, Auckland 1023, New Zealand; a.mcdouall@auckland.ac.nz (A.M.); j.dean@auckland.ac.nz (J.M.D.)

**Keywords:** hypoxic–ischemic encephalopathy, inflammasome, retina, pharmacotherapy, visual impairment, pediatrics

## Abstract

Perinatal hypoxic–ischemic encephalopathy (HIE) is a condition resulting from oxygen deprivation around the time of birth and may be associated with death, brain damage, and disability. Alongside this, studies have shown that HIE may result in visual impairment. Previously, this was thought to be due to damage to the visual pathways in the brain, in a condition known as cerebral visual impairment. However, recent studies suggest that direct injury to the retina may occur after HIE. Of note, the nucleotide-binding domain, leucine-rich-containing family, and pyrin domain-containing-3 (NLRP3) inflammasome is thought to play a role in perpetuating inflammatory damage in the brain after hypoxia–ischemia (HI). As such, this study aimed to characterize retinal inflammation and the role of the NLRP3 inflammasome after HI using a modified Rice-Vannucci model in postnatal day 10 (P10) rat. Eighteen Sprague-Dawley rats were allocated evenly to three groups. Two groups received surgery to ligate the right common-carotid artery and induce HI, while another group received only sham surgery. Rats exposed to HI received subcutaneous injections of tonabersat (HI + Ton) or saline (HI + vehicle) at 1, 24 and 48 h after HI, and were culled at P17 for analysis. The results showed that the protein expression of GFAP, Iba-1, NLRP3, caspase-1 and connexin43 increased in the retina at 7 d after HI-vehicle compared with sham surgery, much more so in the ipsilateral = than the contralateral retina. Furthermore, = inflammasome components NLRP3, cleaved caspase-1 and connexin43 were significantly upregulated in the ipsilateral retina following HI-vehicle compared to the sham surgery group. Treatment with a connexin43 hemichannel blocker, tonabersat, significantly decreased the expression of the inflammasome markers, cleaved caspase-1 and connexin43, and diminished Iba-1+ cell infiltration in the ipsilateral retina. These findings suggest that direct retinal damage and inflammation may occur after HI. Furthermore, these inflammatory changes are likely mediated and propagated by activation of the NLRP3 inflammasome. Importantly, inhibition of the inflammasome by tonabersat may be able to inhibit retinal inflammation and damage, potentially preventing visual impairment after HI. Further investigation in humans is required to determine the efficacy of tonabersat in treating hypoxic–ischemic injuries to the brain and eye.

## 1. Introduction

Perinatal hypoxic–ischemic encephalopathy (HIE) occurs due to a reduction in oxygen and blood supply before, during or shortly after birth. HIE affects 0.1–0.8% of children in the perinatal period and may be associated with irreversible damage to the brain and central nervous system [1,2]. HIE often results in cerebral palsy, visual impairment, developmental delay, cognitive impairment, epilepsy or even death, which can significantly impact the quality of life for the individual and the family involved [3,4,5,6].

The causes of HIE are various and may include placental abruption, cord prolapse, placenta previa, maternal hypotension, fetal distress, congenital heart or lung malformations, post-delivery trauma, infections or cardiorespiratory failure [7,8,9,10,11]. Many clinical and preclinical studies have characterized the evolution and pattern of brain injury associated with the motor and cognitive deficits that result from perinatal hypoxia–ischemia (HI) [12,13,14]. Interestingly, visual impairment has also been commonly reported in children with HIE, but this is less understood [15,16,17,18]. Previously, this was thought to be due to direct damage to neurons in the visual pathway within the brain, also known as cerebral visual impairment (CVI) [19,20,21,22]. However, recent investigations now suggest that this might not be the case, and that the retina may be directly impaired in individuals with HIE (termed ocular visual impairment [OVI]). Studies by Zaitoun et al. found that the retinal neurovascular integrity may be damaged in mouse models of HI injury [23,24]. Clinical studies have also found that many newborns with HIE have retinal hemorrhages, retinal thinning, cystoid macular oedema, maculopathy and subretinal fluid [23,25,26,27]. However, the exact pathophysiology and molecular pathways leading to retinal damage during HIE are currently unclear.

The nucleotide-binding domain, leucine-rich-containing family, and pyrin domain-containing-3 (NLRP3) inflammasome has been implicated in the pathophysiology of tissue inflammation following several forms of ischemic–reperfusion injury, including those of neurovascular etiologies such as strokes [28]. The NLRP3 inflammasome is a multimeric protein complex and is thought to contribute to the initiation and propagation of sterile inflammation after perinatal brain injury and in several other ocular and systemic diseases [29,30]. Activation of the NLRP3 inflammasome is thought to be a two-step process involving a priming and an activation phase [29]. During priming, pathogen-associated molecular patterns (PAMPs), such as bacterial toxins or proteins, or danger-associated molecular patterns (DAMPs), such as reactive oxygen species (ROS), urate or cytoplasmic proteins from cells, bind to toll-like receptors on the cell surface [29,31]. This causes activation of the nuclear factor kappa-B (NF-κB) pathway and increases transcription of NLRP3 protein and inactive pro-inflammatory cytokines pro-interleukin-1-beta (pro-IL-1β) and pro-interleukin-18 (pro-IL-18) [29,31]. A subsequent activation signal, such as extracellular adenosine triphosphate (ATP), potassium, increased intracellular calcium levels or ROS production, allows for the oligomerization of NLRP3 with pro-caspase-1 and apoptosis-associated speck-like protein (ASC) to form a fully functional inflammasome complex [31]. This inflammasome complex allows pro-caspase-1 to be cleaved into functional caspase-1, which can now cleave pro-IL-1β and pro-IL-18 into their active forms (IL-1β and IL-18, respectively) [29,31]. These cytokines are then secreted into the extracellular space, where they can further contribute to and amplify inflammation. Activation of caspase-1 can also contribute to cell death through pyroptosis [29,31].

Several studies have attempted to limit tissue inflammation by blocking inflammasome activation. One target that is of interest is connexin43 hemichannels. Under normal conditions, connexin43 hemichannels typically dock together to form gap junctions [32]. However, under cellular stress, undocked connexin43 hemichannels open to t release ATP into the extracellular space. Extracellular ATP can activate the NLRP3 inflammasome in a paracrine and autocrine manner, leading to amplified inflammation [32]. With relevance to this study, a recent report by McDouall et al. found that tonabersat, a connexin43 hemichannel inhibitor, may reduce the severity of HI-induced cerebral volume loss and neuronal death within the developing rat brain [33]. Tonabersat, an orally available connexin43 hemichannel blocker and a small molecule benzopyran derivative, has been suggested to play a role in inhibiting inflammasome-induced inflammation in several inflammatory diseases [34,35]. Tonabersat crosses the blood–brain barrier and was originally developed for the treatment of migraine caused by cortical spreading depression [35]. Notably, tonabersat has been shown to inhibit inflammasome inhibition by preventing connexin43 hemichannel-mediated ATP release. Tonabersat has been suggested to be effective in preventing inflammasome activation in models of disease, including diabetic retinopathy, age-related macular degeneration, multiple sclerosis, mild hypoxic–ischemic injury of the brain, and diabetic kidney disease [33,34,35,36,37,38,39]. As such, this study aimed to determine the retinal inflammatory changes after HI, and whether the NLRP3 inflammasome plays a role in propagating retinal damage, using a modified Rice-Vanucci model of perinatal HI brain injury in the rat [40]. Furthermore, the role of tonabersat in ameliorating retinal inflammation after HI was investigated.

## 2. Results

### 2.1. Tonabersat Reduces Iba-1+ Cell Infiltration in the Ipsilateral Eye After HI

**Figure 1:**
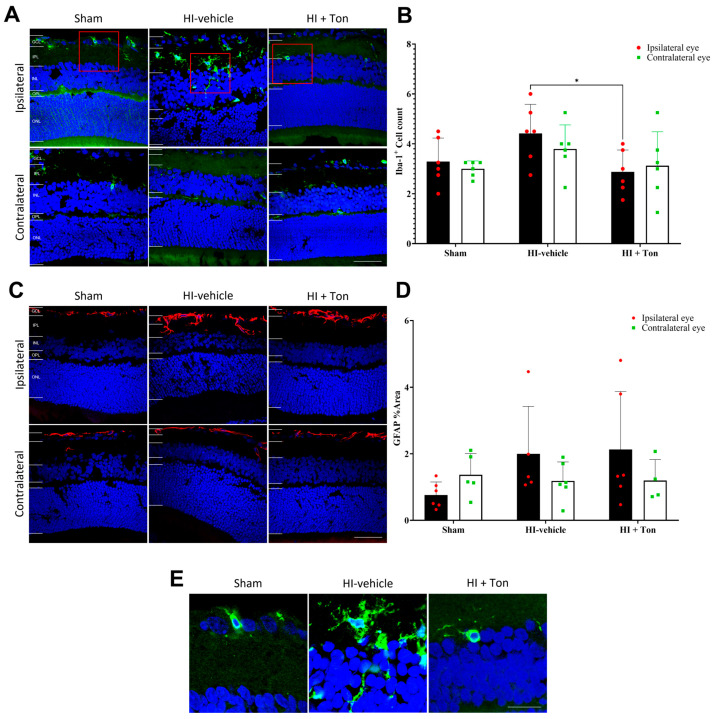
Iba-1+ cell infiltration may be increased in the ipsilateral retina after unilateral occlusion of the common carotid artery. (**A**) Immunohistochemical images showing Iba-1+ cells (green) in the ipsilateral and contralateral eye after carotid occlusion compared with the sham group. Red boxes highlight regions of interest containing Iba-1+ cells, which are zoomed-in within (**E**). There were no differences in Iba-1+ cell infiltration between the ipsilateral and contralateral eyes or between the sham and HI groups. Tonabersat treatment reduced Iba-1+ cell infiltration in the ipsilateral eye following unilateral carotid occlusion. (**B**) Quantification of Iba-1+ cells in the whole retina showed no change in Iba-1+ cell infiltration after carotid occlusion compared with the sham group but was significantly reduced by tonabersat treatment in the ipsilateral eye. (**C**) Immunohistochemical images showing that GFAP (red) expression is increased the most in the ipsilateral eye after HI, which is reversed by tonabersat treatment. (**D**) Quantification of GFAP expression in the whole retina showed HI resulted in no statistically significant upregulation of GFAP compared to sham. (**E**) Zoomed-in images (3x) of Iba-1+ cells from the ipsilateral eye in (**A**). HI = hypoxic–ischemic, HI-vehicle = administration of only vehicle (saline) after hypoxic–ischemic injury, HI + Ton = tonabersat administration after hypoxic–ischemic injury, GCL = ganglion cell layer, IPL = inner plexiform layer, INL = inner nuclear layer, OPL = outer plexiform layer, ONL = outer nuclear layer. Statistical analysis was carried out for Iba-1+ cells and GFAP expression in the entire retina with a two-way ANOVA with a post hoc Šidák’s and Tukey’s multiple comparisons test. *n* = 18. * *p* < 0.05. Green staining is representative of Iba-1+ cells, while blue staining is representative of 4′,6-diamidino-2-phenylindole (DAPI) nuclear DNA staining. Scale bars are provided in the bottom-right panels in (**A**,**C**), which represent 50 µm. The scale bar in the right panel of (**E**) represents 20 µm.

There was no change in Iba-1+ cell infiltration within the ipsilateral eye of the HI group treated only with the saline vehicle (HI-vehicle) compared with the sham group (mean difference: 1.125, 95% CI of difference: −0.3226 to 2.573) (Figure 1A,B). Treatment with tonabersat (HI + Ton group) significantly reduced retinal Iba-1+ cell infiltration in the ipsilateral eye compared with the HI-vehicle group (mean difference: −1.542, 95% CI of difference: −2.989 to −0.09407, *p* = 0.0341). There were no differences in Iba-1+ cell expression between the HI + Ton group and the sham group (mean difference: −0.4167, 95% CI of difference: −1.864 to 1.031).

In the contralateral eye, there were no differences in Iba1+ cell infiltration between the HI-vehicle compared to the sham group (mean difference: 0.7917, 95% CI of difference: −0.6559 to 2.239), and no change with tonabersat treatment (mean difference: −0.6667, 95% CI of diff: −2.114 to 0.781). Similarly, there were no differences in Iba-1+ cell infiltration in the ipsilateral eye compared with the contralateral eye in the HI-vehicle group (mean difference: 0.6250, 95% CI of diff: −0.8226 to 2.073) and other groups.

### 2.2. GFAP Expression Is Not Significantly Elevated in the Ipsilateral Eye After HI and Is Not Reversed by Tonabersat Treatment

Analysis of GFAP expression suggested that there was no difference in GFAP fluorescence in the ipsilateral eye between the HI-vehicle group and the sham surgery group (mean difference: 1.236, 95% CI of difference: −0.3827 to 2.855, *p* = 0.1754) (Figure 1C,D). Similarly, tonabersat had no effect on GFAP expression compared with HI-vehicle in the ipsilateral eye (mean difference: 0.1343, 95% CI of difference: −1.484 to 1.753, *p* = 0.9954). There were also no differences in GFAP expression in the ipsilateral eye between the HI + Ton group and the sham group (mean difference: 1.370, 95% CI of difference: −0.1731 to 2.914, *p* = 0.0931).

In the contralateral eye, there were no differences in GFAP expression in the HI-vehicle group (mean difference: −0.1835, 95% CI of diff: −1.802 to 1.435, *p* = 0.9886) or the HI + Ton group (mean difference: 0.1712, 95% CI of diff: −1.964 to 1.622, *p* = 0.9931) compared with the sham group (Figure 1C,D). Although GFAP expression appeared to be grossly increased on immunohistochemical images in the ipsilateral eye compared with the contralateral eye in the HI-vehicle (mean difference: 0.8119, 95% CI of diff: −0.8069 to 2.431, *p* = 0.5106) and HI + Ton group (mean difference: 0.9338, 95% CI of diff: −0.7918 to 2.659, *p* = 0.4469), this was not statistically significant.

### 2.3. NLRP3 Is Significantly Increased After HI and May Be Reversed by Tonabersat Treatment

**Figure 2:**
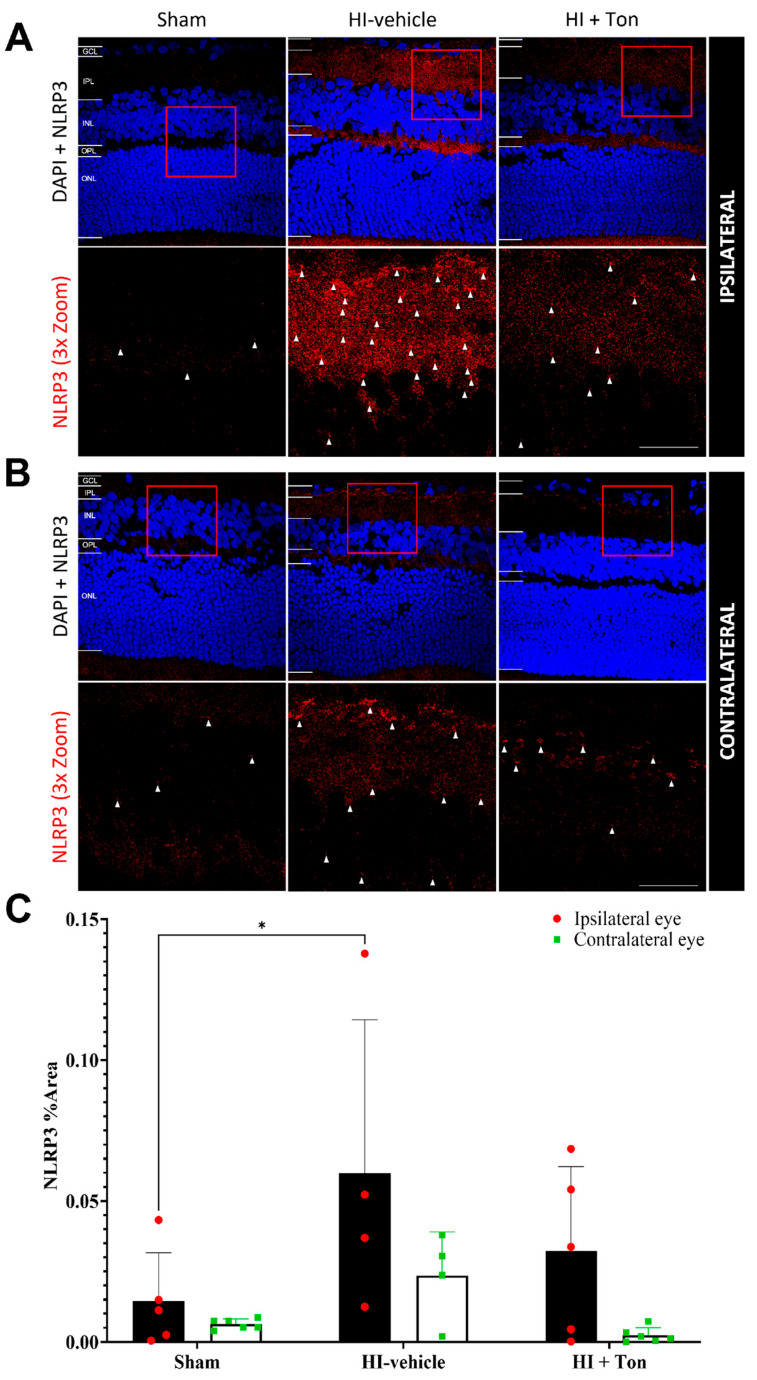
NLRP3 expression was significantly increased in the ipsilateral retina after unilateral occlusion of the common carotid artery. (**A**) Immunohistochemical images from the ipsilateral retina showed that NLRP3 expression (red) was increased in the ipsilateral eye in the HI-vehicle group compared to the sham surgery group. Treatment with tonabersat appeared to decrease NLRP3 expression. (**B**) Immunohistochemical images from the contralateral retina showed that NLRP3 expression (red) was slightly increased in the HI-vehicle group compared to the sham surgery group. However, this was not greater than NLRP3 expression in the ipsilateral retina. Treatment with tonabersat appeared to decrease NLRP3 expression in the contralateral eye after HI. (**C**) Quantification of NLRP3 expression in the whole retina showed that HI resulted in a statistically significant increase in NLRP3 expression in the HI-vehicle group compared to the sham surgery group within the ipsilateral eye (*p* = 0.0325). This increase was reduced by tonabersat treatment in the HI + Ton group. HI + Ton = tonabersat administration after hypoxic–ischemic injury, HI-vehicle = administration of only vehicle (saline) after hypoxic–ischemic injury, GCL = ganglion cell layer, IPL = inner plexiform layer, INL = inner nuclear layer, OPL = outer plexiform layer, ONL = outer nuclear layer. Scale bars represent 20 µm. Statistical analysis was carried out for NLRP3 expression in the entire retina with a two-way ANOVA with a post hoc Šidák’s and Tukey’s multiple comparisons test. *n* = 18. * *p* < 0.05. Red staining is representative of NLRP3 expression, while blue staining is representative of 4′,6-diamidino-2-phenylindole (DAPI) nuclear DNA staining. Red boxes highlight regions of interest which were zoomed-in upon to produce the image immediately below. White arrows highlight regions of interest with enhanced NLRP3 expression. Scale bars are provided in the bottom-right panels in (**A**,**B**), which represent approximately 20 µm.

Quantifying the % area of NLRP3 labelling in the ipsilateral eye showed that NLRP3 expression was significantly increased in the HI-vehicle group compared with the sham group (mean difference: 0.04537, 95% CI of diff: 0.003150 to 0.08759, *p* = 0.0325) (Figure 2A–C). There was no effect of Tonabersat on NLRP3 expression following HI in the ipsilateral eye (mean difference: −0.02765, 95% CI of diff: −0.06987 to 0.01457) (Figure 2A–C).

In the contralateral eye, there were no differences in NLRP3 expression in the HI-vehicle group compared with the sham surgery group (mean difference: 0.01720, 95% CI of diff: −0.02343 to 0.05782, *p* = 0.6393) (Figure 2C). There was also no effect of Tonabersat (HI + Ton) on NLRP3 expression following HI in the contralateral eye’s mean difference: −0.02118, 95% CI of diff: −0.06181 to 0.01945, *p* = 0.4754) (Figure 2C). Furthermore, NLRP3 expression appeared to be generally elevated in the ipsilateral eye compared with the contralateral eye after HI, although this was not statistically significant (Figure 2A–C).

### 2.4. Caspase-1 Is Significantly Increased in HI and Is Reversed by Tonabersat Treatment in the Ipsilateral Eye

**Figure 3:**
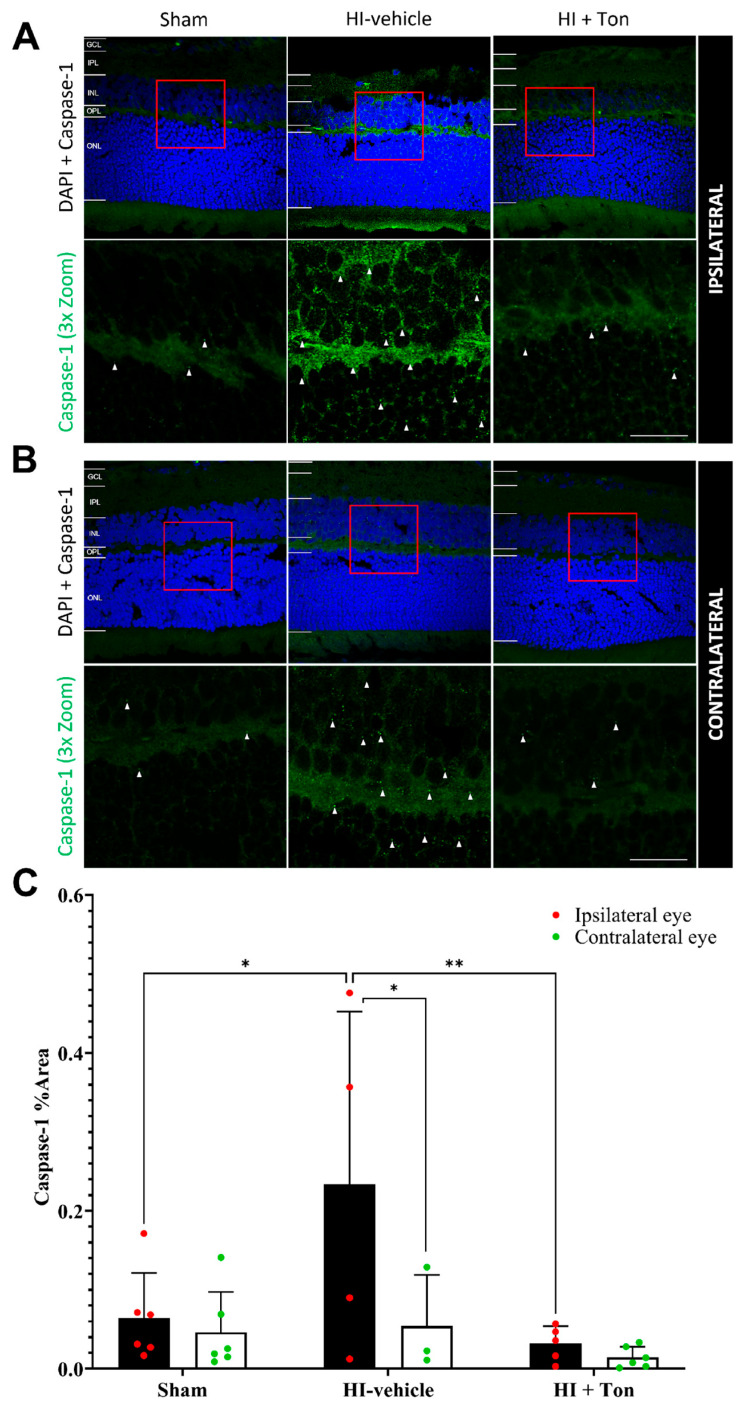
Cleaved caspase-1 expression was significantly increased in the ipsilateral retina after unilateral occlusion of the common carotid artery. (**A**) Immunohistochemical images from the ipsilateral retina showed that cleaved caspase-1 expression (green) was increased in the ipsilateral eye in the HI-vehicle group compared to the sham surgery group. Treatment with tonabersat appeared to decrease cleaved caspase-1 expression. (**B**) Immunohistochemical images from the contralateral retina showed that cleaved caspase-1 expression increased slightly in the HI-vehicle group compared to the sham surgery group. However, this was not greater than cleaved caspase-1 expression (green) in the ipsilateral retina. Treatment with tonabersat appeared to decrease cleaved caspase-1 expression in the contralateral eye after HI (HI + Ton). (**C**) Quantification of cleaved caspase-1 expression in the whole retina showed that HI resulted in significantly greater cleaved caspase-1 expression in the HI-vehicle group compared to the sham surgery group (*p* = 0.0186). Treatment with tonabersat following HI (HI + Ton) significantly reduced cleaved caspase-1 expression in the ipsilateral eye (*p* = 0.0064). Cleaved caspase-1 expression was significantly higher in the ipsilateral eye compared to the contralateral eye in the HI-vehicle group (*p* = 0.0382). HI + Ton = tonabersat administration after hypoxic–ischemic injury, HI-vehicle = administration of only vehicle (saline) after hypoxic–ischemic injury, GCL = ganglion cell layer, IPL = inner plexiform layer, INL = inner nuclear layer, OPL = outer plexiform layer, ONL = outer nuclear layer. Scale bars represent 20 µm. Statistical analysis was carried out for caspase-1 expression in the entire retina with a two-way ANOVA with a post hoc Šidák’s and Tukey’s multiple comparisons test. *n* = 18. * *p* < 0.05, ** *p* < 0.01. Green staining is representative of cleaved caspase-1 expression, while blue staining is representative of 4′,6-diamidino-2-phenylindole (DAPI) nuclear DNA staining. Red boxes highlight regions of interest which were zoomed-in upon to produce the image immediately below. White arrows highlight regions of interest with cleaved caspase-1 expression. Scale bars are provided in the bottom-right panels of (**A**,**B**), which represent approximately 20 µm.

In the ipsilateral eye, the quantification of the % area covered by caspase-1 in the ipsilateral eye showed that caspase-1 expression was significantly increased in the HI-vehicle group compared with the sham group (mean difference: 0.1694, 95% CI of difference: 0.02439 to 0.3144, *p* = 0.0186) (Figure 3C). Tonabersat treatment significantly reduced caspase-1 expression in the HI + Ton group compared with the HI-vehicle group (mean difference: −0.2020, 95% CI: −0.3527 to −0.05126, *p* = 0.0064). There was no difference in caspase-1 expression between the sham and HI + Ton groups in the ipsilateral eye. These findings were similarly reflected on immunohistochemical images (Figure 3A,B).

Notably, after HI injury, caspase-1 expression was further elevated in the ipsilateral eye compared with the contralateral eye (Figure 3A). This was confirmed by quantifying caspase-1 expression, which showed that expression in the HI-vehicle group was significantly increased in the ipsilateral eye compared with the contralateral eye (mean difference: 0.1796, 95% CI: 0.008041 to 0.3512, *p* = 0.0382). Tonabersat treatment ameliorated this difference, with no difference in caspase-1 expression identified between the ipsilateral and contralateral eyes following drug administration (mean difference: 0.01733, 95% CI: −0.1187 to 0.1534, *p* = 0.9837).

### 2.5. Connexin43 Is Significantly Increased in HI and Is Reversed by Tonabersat Treatment

**Figure 4:**
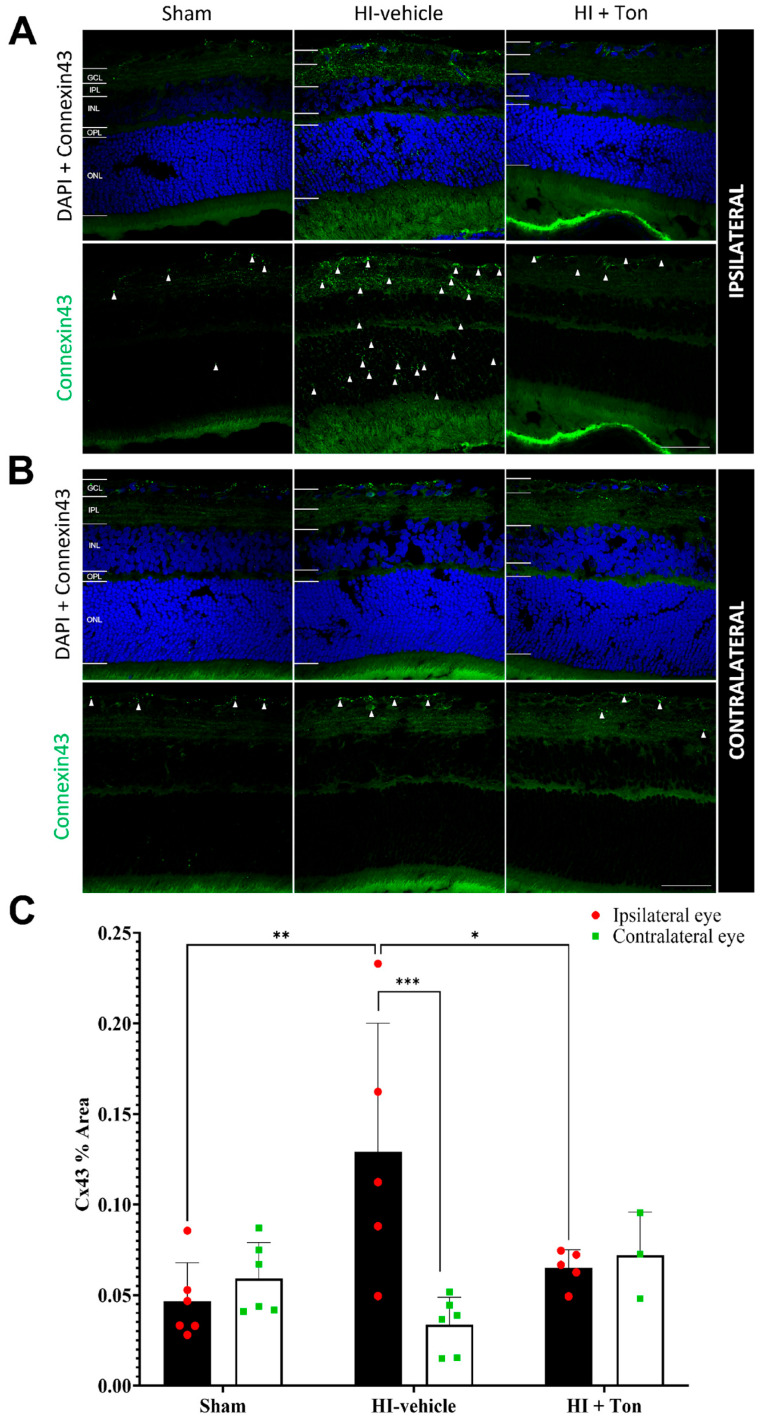
Connexin43 expression was significantly increased in the ipsilateral retina after unilateral occlusion of the common carotid artery. (**A**) Immunohistochemical images from the ipsilateral retina showed that connexin43 expression (green) was increased in the ipsilateral eye in the HI-vehicle group compared to the sham surgery group. Treatment with tonabersat appeared to decrease connexin43 expression. (**B**) Immunohistochemical images from the contralateral retina showed no observable differences in connexin43 expression (green) between groups. (**C**) Quantification of connexin43 expression in the whole retina showed that HI resulted in significantly greater connexin43 expression in the HI-vehicle group compared to the sham surgery group (*p* = 0.0010). Treatment with tonabersat following HI significantly reduced connexin43 expression in the ipsilateral eye (*p* = 0.0151). Connexin43 expression was significantly higher in the ipsilateral eye compared to the contralateral eye in the HI-vehicle group (*p* = 0.0002). HI + Ton = tonabersat administration after hypoxic–ischemic injury, HI-vehicle = administration of only vehicle (saline) after hypoxic–ischemic injury, GCL = ganglion cell layer, IPL = inner plexiform layer, INL = inner nuclear layer, OPL = outer plexiform layer, ONL = outer nuclear layer. Scale bars represent 20 µm. Statistical analysis was carried out for connexin43 expression in the entire retina with a two-way ANOVA with a post hoc Šidák’s and Tukey’s multiple comparisons test. *n* = 18. * *p* < 0.05, ** *p* < 0.01, *** *p* < 0.001. Green staining is representative of connexin43 expression, while blue staining is representative of 4′,6-diamidino-2-phenylindole (DAPI) nuclear DNA staining. White arrows high-light regions of interest with prominent connexin43 expression. Scale bars are provided in the bottom-right panels of (**A**,**B**), which represent 50 µm.

After HI, connexin43 expression was significantly increased in the ipsilateral eye compared with the sham group, particularly within the inner retina, while tonabersat treatment reduced connexin43 expression (Figure 4A). Within the contralateral eye, there was no change in connexin43 expression in the HI-vehicle group compared with the sham control (Figure 4B).

This was confirmed through connexin43 expression in the ipsilateral eye, which showed that the % area covered by connexin43 was significantly increased in the HI-vehicle compared with the sham group (mean difference: 0.08247, 95% CI: 0.03146 to 0.1335, *p* = 0.0010) (Figure 4C). Treatment with tonabersat in the HI + Ton group significantly reduced connexin43 expression compared with the HI-vehicle group (mean difference: −0.06398, 95% CI: −0.1173 to −0.0170, *p* = 0.0151). Tonabersat treatment restored connexin43 levels to baseline levels in the ipsilateral eye, where connexin43 levels were similar between the HI + Ton group and sham groups (mean difference: 0.01849, 95% CI: −0.03252 to 0.06951, *p* = 0.7410).

There were no differences in connexin43 expression between the sham, HI-vehicle and HI + Ton groups in the contralateral eyes. In the HI-vehicle group, connexin43 expression was significantly increased in the ipsilateral eye compared with the contralateral eye (mean difference: 0.09541, 95% CI: 0.04439 to 0.1464, *p* = 0.0002). This difference was ameliorated by the tonabersat treatment, where connexin43 expression was similar between the ipsilateral and contralateral eyes in the HI + Ton group (mean difference: −0.006966, 95% CI: −0.06849 to 0.05456, *p* = 0.9885).

### 2.6. IL-1β Expression Is Significantly Elevated During HI and Is Reversed by Tonabersat Administration

**Figure 5:**
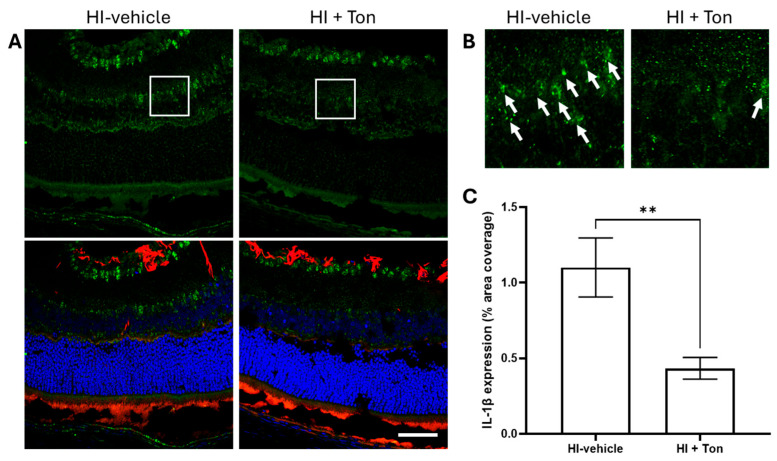
Tonabersat significantly reduced the expression of IL-1β in the retina of rats after unilateral occlusion of the common carotid artery. (**A**) Immunohistochemical images from the retina showed that IL-1β expression (green) was reduced in mice that were administered tonabersat. GFAP expression (red) also appeared to follow this pattern. (**B**) Zoomed-in images (5x) of the retina showing amelioration of IL-1β expression by tonabersat. (**C**) Quantification of IL-1β expression in the whole retina showed that administration of tonabersat to mice resulted in a significantly lower expression of IL-1β in the HI + Ton group compared to the HI + vehicle group (*p* = 0.0052). HI + Ton = tonabersat administration after hypoxic–ischemic injury, HI-vehicle = administration of only vehicle (saline) after hypoxic–ischemic injury. Scale bars represent 20 µm. Statistical analysis was carried out for IL-1β expression in the entire retina with a Student’s *t*-test. *n* = 18. ** *p* < 0.01. Green staining is representative of IL-1β expression, red staining is representative of GFAP expression, while blue staining is representative of 4′,6-diamidino-2-phenylindole (DAPI) nuclear DNA staining. White boxes highlight regions of interest which were zoomed-in upon to produce (**B**). White arrows high-light regions of interest with prominent IL-1β expression. A scale bar is provided in the bottom-right panel of (**A**), which represents 40 µm.

Further investigation showed that IL-1β expression appeared to be raised during HI-injury, which was reversed by tonabersat administration in mice (Figure 5A,B). Quantification of IL-1β expression suggested that this difference was statistically significant (Figure 5C, mean difference: −0.6667, 95% CI: −0.9021 to −0.4313, *p* = 0.0052).

### 2.7. Connexin43 Is Positively Correlated with NLRP3 and Caspase-1 Expression

**Figure 6:**
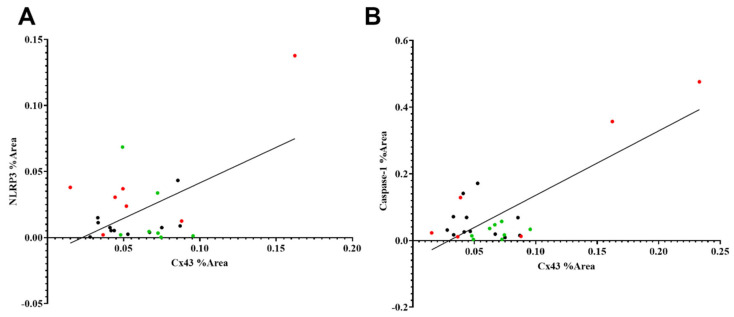
Simple linear regression between the % Area covered by NLRP3, caspase-1 and connexin43. (**A**) % Area covered by NLRP3 and connexin43 have a moderate positive linear relationship (*R*^2^ = 0.2870). The gradient of the line of best fit was significantly non-zero (*p* = 0.0058). (**B**) % Area covered by cleaved caspase-1 and connexin43 have a strong positive linear relationship (*R*^2^ = 0.6037). The gradient of the line of best fit was significantly non-zero (*p* < 0.0001). Equations for lines of best fit are provided in Table 1. Red dots = HI-vehicle, green dots = HI + Ton, black dots = sham surgery.

Connexin43 expression in the rat retina was positively correlated with NLRP3 expression (*R*^2^ = 0.2870, *p* = 0.0058) and caspase-1 expression (*R*^2^ = 0.6037, *p* < 0.0001) (Figure 6A,B). Some rat eyes that underwent HI-vehicle demonstrated relatively higher levels of connexin43 and NLRP3, while rat eyes in the HI + Ton group and sham had lower levels of connexin43 and NLRP3 expression (Figure 5A,B). Equations for lines of best fit are provided in Table 1.

## 3. Discussion

HIE is a serious and devastating condition that may affect infants in the perinatal period, often resulting in irreversible cerebral damage and cerebral palsy [3,4,5,6]. Several studies have suggested that visual impairment may also occur because of HI [15,16,17,18]. While this was previously thought to be due to direct injury to the visual pathway or primary visual cortices in the brain [19,20,21,22], recent investigations have provided evidence for a direct injury to retinal neurons and blood vessels, resulting in visual impairment [23,24].

In the present study, the Rice-Vanucci model of HIE was modified to study retinal changes after neonatal HI injury [40]. Our findings indicate that after HI, it is likely that inflammatory changes consistent with NLRP3 inflammasome activation and more general inflammatory changes occur within the retina. GFAP expression can increase within the retina due to upregulation of Müller cell processes during inflammation, trauma, or stress [41]. Although not statistically significant, our findings indicated that after HI, GFAP may be increased in the ipsilateral eye, suggesting that retinal stress and damage indeed occur. Similarly, during retinal degeneration or inflammation, Iba-1+ cells (activated microglia and macrophages) may invade more deeply into the retina and undergo morphological changes [42].

Importantly, activation of the NLRP3 inflammasome is thought to play a role in the propagation and pathogenesis of sterile retinal inflammation, particularly during ocular diseases that involve retinal vasculature, such as proliferative diabetic retinopathy [43,44]. Our findings suggested that NLRP3 and caspase-1 protein expression significantly increased within the retina after HI within the ipsilateral eye. Connexin43, a hemichannel that is thought to activate the NLRP3 inflammasome through the release of extracellular ATP, was also significantly increased in the retina of rats after HI induced by unilateral carotid occlusion. These results suggest that retinal inflammation may be perpetuated by activating the NLRP3 inflammasome, contributing to retinal damage and visual impairment after HI.

Analysis with simple linear regression identified that the retinal expression of NLRP3 and caspase-1 was positively correlated with connexin43 expression. Notably, cleaved caspase-1 had a stronger association with connexin43 compared with NLRP3. This is likely because NLRP3 is elevated during both inflammasome priming and activation. By contrast, cleaved caspase-1 only increases during inflammasome activation [29,31], which can be driven by connexin43-mediated ATP release. On the other hand, although GFAP expression was not significantly reduced following tonabersat treatment, some studies have suggested that after experiencing noxious stimuli, GFAP may be persistently upregulated for some time, even after the removal of the initial stimulus [45,46]. Altogether, our findings suggest that the inflammasome is upregulated after HI injury in the ipsilateral retina and likely contributes to inflammation after HI injury.

The treatment of rats after HI with tonabersat, an inhibitor of connexin43 hemichannels, yielded promising results. Rats that received tonabersat showed significantly reduced levels of caspase-1 and connexin43 in the retina. Furthermore, a key downstream cytokine produced as a result of inflammasome activation, IL-1β, was significantly reduced by tonabersat administration. This indicates that tonabersat effectively inhibited NLRP3 inflammasome-mediated inflammation of the retina after HI. Although our results suggested that tonabersat had a non-significant effect on the levels of NLRP3 protein, its effects on cleaved caspase 1 and IL-1β suggest an ability to reduced inflammasome activation. Interestingly, tonabersat also significantly reduced Iba-1+ cell prevalence and activation in the retina of HI rats, suggesting that retinal damage and inflammation after HI may be prevented by tonabersat administration following HI injury.

Overall, our findings suggest that in this animal model of HI brain injury, the ipsilateral retina is commonly affected and displays the elevation of general inflammatory markers. Furthermore, the NLRP3 inflammasome is activated and contributes to inflammatory damage to the retina. These findings align with other studies investigating NLRP3 expression within the brain parenchyma. Several reports have demonstrated that NLRP3, caspase-1, and IL-1β are upregulated in the brain after HI [47,48,49,50,51,52,53,54]. In fact, specific inhibition of NLRP3 by MCC950, ASC knockout, and other non-specific NLRP3 inhibitors alleviated the severity of brain injury in models of neonatal HI, suggesting that the NLRP3 inflammasome plays an important role [48,51,52,53,54,55].

Akin to this, we found that NLRP3 was also upregulated in the retina after HI, and that these changes were reversed by administering an upstream inflammasome blocker, tonabersat, which acts upon connexin43 hemichannels. A recent investigation by McDouall et al., which also used the modified Rice-Vanucci model, found that tonabersat was efficacious in preventing neural tissue volume loss, improving neuronal survival, and preventing Iba1+ cell infiltration, especially within the hippocampus and the corpus callosum [33]. Taken together, this suggests that tonabersat may be an efficacious clinical therapy for HIE, preventing retinal damage and vision loss by limiting neural tissue death and inflammation through blocking the NLRP3 inflammasome pathway.

### Further Considerations

One important observation was that data variability in the HI-vehicle group was much higher than in the HI + Ton and sham groups. This phenomenon has been well-described by various other studies utilizing the Rice-Vanucci HI model [56]. Several sources of variability have been proposed, including animal strain, litter size, duration of anaesthesia, hypothermia, maternal diet, and blood sugar levels [56]. Within the context of this study, one prominent source of variation may be collateral blood flow during common carotid artery (CCA) occlusion. Within rats and humans, the ophthalmic artery is a distal branch of the internal carotid artery, which is supplied proximally by the CCA under normal conditions [57]. During CCA occlusion, the circle of Willis is the primary source of collateral blood flow that can maintain ICA patency [58]. Alongside this, Edwards et al. demonstrated that in the Rice-Vanucci model of HI, collateral blood flow from an anastomosis between the vertebral artery and the external carotid artery may still allow for some patency and perfusion of the internal carotid artery through retrograde blood flow even during unilateral CCA occlusion [56,59]. In fact, Wang et al. also described four of many complex collateral circulation pathways that may be able to maintain patency in the ipsilateral ICA despite unilateral CCA occlusion [58,60]. This may mean that although there may be CCA occlusion, some blood flow within the ophthalmic artery may be preserved.

Furthermore, several anatomic variations in the major cerebral arteries of humans and animals may exist between different individuals, resulting in varying innate abilities to compensate for CCA occlusion [58,61,62,63,64,65]. Some sources have even indicated that anomalies of arteries linked to the circle of Willis may be found in as many as 30% of human individuals [58,66]. Additionally, angiogenesis in the brain is poorly understood, and new collateral channels may develop after birth in an unpredictable manner [58]. These may cause inconsistent levels of collateral blood flow between study animals, which may have also contributed to the variability observed in this study [56].

Other limitations of this study include the lack of area-specific data within the retina, the unavailability of data for other relevant proteins such as ASC, and that our data were limited to one time point. Additionally, quantification of extracellular adenosine triphosphate (ATP) released by connexin43 hemichannels would have further supported our claims and improved the impact of this study. However, in vivo quantification of ATP is very challenging and poses difficulties due to the highly localised nature of ATP, owing to its primarily autocrine and paracrine activity. Future investigations that include other inflammasome proteins, data from various regions of the retina and serial time points would provide more insight into the evolution of the retinal inflammatory response after HIE and help elucidate the role of the NLRP3 inflammasome in HIE further.

## 4. Materials and Methods

### 4.1. Animal Husbandry

All experiments were conducted with ethics approval and per the regulations of the University of Auckland Animal Ethics Committee (approval number: R2020). A total of 24 healthy male and female Sprague Dawley rat pups, from 3 litters at postnatal day 10 (P10), were housed with their dams under standard laboratory conditions at 23 ± 2 °C at the Vernon Jansen Unit (VJU) Animal Facility, University of Auckland. A twelve-hour light–dark cycle was maintained throughout the housing.

### 4.2. Inducing HI Injury to the Retina

A modified version of the Rice-Vanucci model was utilized to model perinatal hypoxic–ischaemic encephalopathy as previously described by McDouall [33]. At P10, pups were anaesthetized by inhalation of carbon dioxide. Any fur was removed from the surgical site with a sterile razor blade. The right neck was prepped with ethanol. Sharp and blunt dissection was utilized to expose the right carotid artery. The right carotid artery was cauterized, and the wound was appropriately closed. In the control group (Sham, *n* = 6), sham surgery was conducted to expose the carotid artery but without any ligation of the carotid artery. Pups did not receive any post-surgical antibiotics or pain relief.

Following surgery, pups were housed with the dam for 1 h to allow for recovery from anaesthesia. Rats were then exposed to hypoxia (8% oxygen, 92% nitrogen) for 90 min in a specifically designed hypoxic chamber at 35 ± 0.5 °C.

Pups who underwent unilateral carotid artery occlusion were randomly allocated to be administered subcutaneous saline (Sigma-Aldrich; St. Louis, MO, USA) (HI group, *n* = 6) or tonabersat 2 mg/kg (MedChemExpress; Monmouth Junction, NJ, USA) (HI + Ton group, *n* = 6), dissolved in the vehicle 2-hydroxypropyl-beta-cyclodextrin polyethylene glycol-400 [PEG] (Sigma-Aldrich; St. Louis, MO, USA), or the vehicle alone (*n* = 6) at 1, 24 and 48 h after hypoxia. For the first 24 h, the cage housing the pups and dam was placed on a heating pad at 36 °C.

At P17, rats were euthanized with an overdose of carbon dioxide and sacrificed. Both eye globes were collected and fixed in 10% formalin for 24 h, then transferred into 70% ethanol before being paraffin-embedded. Samples were then sectioned into 5 µm slices using a microtome and mounted onto glass slides for immunohistochemical analysis.

### 4.3. Immunohistochemistry

Eye globe sections were washed in phosphate-buffered saline (PBS) five times for 5 min, then blocked in PBS containing 0.1% Triton and 10% normal horse serum for 1 h at room temperature. Following blocking, slides were dried and incubated with the primary antibodies for GFAP, connexin43, Iba-1, caspase-1 and NLRP3 at 4 °C for 24 h (Table 2). The next day, slides were washed in PBS 5 times for 5 min and incubated with secondary antibodies for 2 h at room temperature in the dark. Cell nuclei were stained blue using 4′,6-diamidino-2-phenylindole (DAPI) (1:200; D9542; Sigma-Aldrich, USA). After incubation, sections were washed in the dark with phosphate-buffered saline (PBS) 5 times for 5 min. Slides were then mounted using an anti-fade reagent (Citifluor™; Hatfield, PA, USA), and coverslips were sealed with nail polish.

### 4.4. Confocal Image Acquisition and Analysis

Immunohistochemistry images were obtained using an FV1000 confocal laser scanning microscope (Olympus, Tokyo, Japan). Images were processed using FV_10 ASW 4.2 Viewer software and were quantified using ImageJ 1.53 software (National Institutes of Health; Bethesda, MD, USA). Laser power, gain, and offset parameters were kept constant for each biomarker to allow for a fair comparison between groups. The researcher was masked during image acquisition to minimize bias further. During quantification, all images were converted into binary images, and equal low threshold values were applied to all images for each marker. Area fraction (% area) was calculated for each marker. Quantification was carried out for the whole retina from the inner limiting membrane to the external limiting membrane. Iba-1 cell counts were carried out manually by a masked researcher. Quantification of each marker was repeated in four separate regions of the retina and averaged for each mouse.

### 4.5. Statistical Analysis

Data were analyzed using GraphPad Prism 9.3.1 software (GraphPad; Boston, MA,USA). Fluorescent quantification values were presented as mean and standard deviations. Protein quantification was carried out in the entire retina for both eyes. Two-way ANOVA tests with post hoc Tukey’s and Šidák’s multiple comparisons test to analyse for differences between ipsilateral and contralateral eyes and between treatment groups. The assumption of sphericity was met, as independent test subjects were used for each of the three exposure groups, and there were only two levels for the laterality of the eyes. Normality was assessed with manual inspection of Q–Q plots. A *p*-value of less than 0.05 was considered statistically significant in all analyses.

## 5. Conclusions

HIE is a serious condition that may affect children in the perinatal period, which may result in irreversible damage to the brain. Visual impairment is also commonly associated with HI and was previously thought to be due to damage to the primary visual cortices. However, our findings suggest that the retina may be directly affected after HI, and it demonstrates signs of acute inflammation after HI injury. Notably, the NLRP3 inflammasome may mediate retinal inflammation after HI. Upstream pharmacological inhibition of the inflammasome with connexin43 hemichannel blockers such as tonabersat may reduce retinal inflammation and the extent of visual loss after HI. However, further testing with animals and clinical trials in humans is required to validate the efficacy of tonabersat in treating the functional deficits observed after HIE.

## Figures and Tables

**Table 1:** Simple linear regression values for connexin43 against NLRP3 and cleaved caspase-1.

Parameters	*R*^2^ Value	Equation for the Line of Best Fit	Gradient Significantly Non-Zero (*p* < 0.05)?
NLRP3 vs. Cx43	0.2870	y=0.5273x−0.01232	*p* = 0.0058
Caspase-1 vs. Cx43	0.6037	y=1.932x−0.05764	*p* < 0.0001

**Table 2:** Details of antibodies utilized in this study.

Antibodies	Origin	Antibody Type	IHC Working Dilution	Catalogue Number	Company
GFAP-Cy3	Mouse monoclonal	Primary	1:1000	C9205	Sigma-Aldrich
Connexin43	Rabbit polyclonal	Primary	1:2000	C6219	Sigma-Aldrich
Iba-1	Rabbit polyclonal	Primary	1:2000	ab178846	Abcam; Cambridge, UK
Cleaved caspase-1	Rabbit polyclonal	Primary	1:500	MA5-38099	Invitrogen; Carlsbad, CA, USA
NLRP3	Goat polyclonal	Primary	1:500	ab4207	Abcam
Donkey anti-Rabbit Alex 488	Secondary Donkey polyclonal	Secondary	1:500	A-21206	Invitrogen
Donkey anti-Goat Cy3	Secondary Donkey polyclonal	Secondary	1:500	705-165-147	Jackson Immuno Research; Cambridge, UK
Goat anti-Rabbit Alex Fluor 488	Secondary Goat polyclonal	Secondary	1:500	A-11034	Invitrogen

## Data Availability

All data generated or analysed during this study are included in the published article.
